# Pathway level analysis of gene expression using singular value decomposition

**DOI:** 10.1186/1471-2105-6-225

**Published:** 2005-09-12

**Authors:** John Tomfohr, Jun Lu, Thomas B Kepler

**Affiliations:** 1 Department of Biostatistics and Bioinformatics and Center for Bioinformatics and Computational Biology, Institute for Genome Sciences and Policy, Duke University, Durham, North Carolina 27708, USA

## Abstract

**Background:**

A promising direction in the analysis of gene expression focuses on the changes in expression of specific predefined sets of genes that are known in advance to be related (e.g., genes coding for proteins involved in cellular pathways or complexes). Such an analysis can reveal features that are not easily visible from the variations in the individual genes and can lead to a picture of expression that is more biologically transparent and accessible to interpretation. In this article, we present a new method of this kind that operates by quantifying the level of 'activity' of each pathway in different samples. The activity levels, which are derived from singular value decompositions, form the basis for statistical comparisons and other applications.

**Results:**

We demonstrate our approach using expression data from a study of type 2 diabetes and another of the influence of cigarette smoke on gene expression in airway epithelia. A number of interesting pathways are identified in comparisons between smokers and non-smokers including ones related to nicotine metabolism, mucus production, and glutathione metabolism. A comparison with results from the related approach, 'gene-set enrichment analysis', is also provided.

**Conclusion:**

Our method offers a flexible basis for identifying differentially expressed pathways from gene expression data. The results of a pathway-based analysis can be complementary to those obtained from one more focused on individual genes. A web program PLAGE (Pathway Level Analysis of Gene Expression) for performing the kinds of analyses described here is accessible at .

## Background

Gene expression microarrays provide a snapshot of the expression levels of thousands of genes within a cell or tissue sample. A persistent challenge is to interpret this data: to identify key genes or patterns of expression associated with some condition and so to gain valuable clues about the biological processes related to that condition.

While a variety of methods have been developed to identify significant changes in the expression of individual genes [1-4], another useful perspective can be gained by viewing expression data at the level of groups of related genes. One approach along these lines identifies similarities, such as shared pathways or GO annotations [5], between genes previously identified in an individual gene analysis [6,7]. A potential problem is that this approach relies on the individual genes within a category of interest to stand out. Modest but consistent changes in the expression of a group of related genes could be missed if relatively few of the individual genes appear significant.

A promising alternative focuses at the outset on identifying significantly differently expressed groups of genes from a collection of predefined sets of genes (e.g., pathways and complexes) [8,9]. The usefulness of such an approach was strikingly demonstrated by Mootha *et al*. [9] in a study of gene expression profiles of muscle in type 2 diabetics (DM2). As reported by them, no single gene showed up as significant in a comparison between DM2s and subjects with normal glucose tolerance (NGT). Their 'gene-set enrichment analysis' (GSEA), however, uncovered a set of genes involved in oxidative phosphorylation as being significantly downregulated in DM2 vs. NGT.

In this article we present a new pathway based approach to the analysis of gene expression that, while similar in spirit to GSEA, has a number of potential advantages. Briefly, GSEA involves ranking all the genes (for example, by significance level in a two-group comparison) and then calculating an 'enrichment score' (ES) for each pathway that depends on the rankings of its member genes. Our method instead begins by translating gene expression levels into pathway 'activity' levels, which are derived from singular value decompositions (SVD). The activity levels are used for making comparisons and in general can be used in the same kinds of applications as gene expression levels.

We demonstrate the approach using the same expression data analyzed by Mootha *et al*. [9] in their study of type 2 diabetes, and also with expression data from airway epithelia of smokers and non-smokers [10]. Our analysis leads us to conclusions similar to those obtained using GSEA in the diabetes set, but overall appears to perform better in identifying differentially expressed pathways in comparisons between smokers and non-smokers.

The results presented in this article, including statistics for pathways and colormaps of expression profiles, were obtained using a web program we have developed called PLAGE (Pathway Level Analysis of Gene Expression) [11].

## Results and discussion

### Outline of the method

In the next two sections we analyze gene expression data from skeletal muscle of type 2 diabetics and airway epithelia of different types of smokers. Here we give a brief overview of our approach. A more detailed description is given in the Methods section.

**Figure 1:**
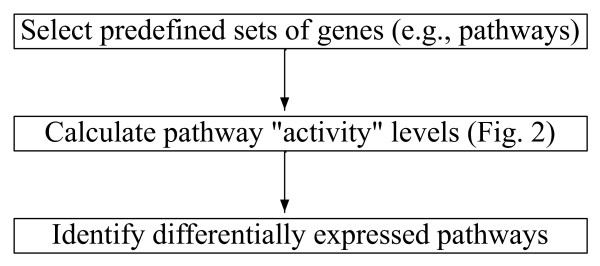
Outline of pathway level analysis of gene expression.

The method is outlined in Fig. 1. The analysis is based on a predefined collection of pathways (e.g., sets of genes coding for proteins involved in specific metabolic or signaling pathways). We use a collection of about 400 pathways obtained from the KEGG (Kyoto Encyclopedia of Genes and Genomes) and Biocarta websites [12,13].

The main goal is to determine, based on the gene expression data, which (if any) of the pathways are associated with some variable of interest such as disease status. To address this, we start by calculating activity levels for each pathway within the samples (in this article, each sample is the gene expression profile in a tissue sample from one individual).

**Figure 2:**
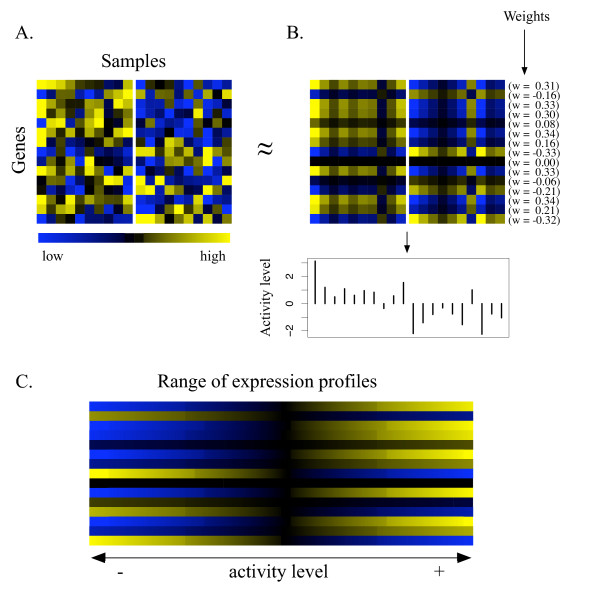
**Pathway activity levels**. Schematic illustration of our approach to quantifying the activity level of a pathway. (*A*) A colormap of the expression levels for the genes in a hypothetical pathway after standardizing the expression levels to have zero mean and unit variance over samples. This represents the matrix *Y *described in the text. (*B*) The main component of the variation in the expression matrix depicted in (*A*). This representation is determined by the activity levels **c **and weights **w **(see Methods) associated with the first metagene in the singular value decomposition (SVD) of *Y *. The activity level in a sample (one column of the expression matrix) can be thought of as specifying a location in the range of expression profiles shown in (*C*). Positive activity levels here indicate relatively high (low) expression for genes with positive (negative) weight. For example, the expression profile (column) furthest to the left in the expression matrix is in the high positive region of the range of expression profiles. The colormaps in (*A*) and (*B*) show the samples divided into two hypothetical groups (e.g., case samples and control samples). We note, however, that the matrix *Y *contains expression values for all samples: the activity levels are determined by performing SVD using expression data from all samples without regard to how the samples are classified.

We define the activity level in terms of the first eigenvector, 'metagene', in the singular value decomposition (SVD) of the matrix of expression levels *Y *(Fig. 2A). The expression matrix is restricted, however, to include only those genes within one predefined pathway at a time. This restriction is one of the main differences from previous applications of SVD (e.g., [14,15]) to gene expression analysis.

As a gene expression value represents the level of expression of a gene in some sample, the activity level represents the 'level' of the first metagene in a sample. The first metagene is simply a vector of weights, one weight for each gene, and a positive activity level indicates the relatively high (low) expression of genes with positive (negative) weight; a negative activity level indicates the reverse. The activity level in a given sample can be thought of as specifying the position of an expression profile (one column of the expression matrix) in a range of possible profiles as shown in Fig. 2C.

The main motivation for using the first metagene from SVD to define the activity level is that the weights (first metagene) and associated activity levels together capture the main component of the variation in the full expression matrix *Y *(Fig. 2B depicts the main component of the variation for the expression matrix in Fig. 2A). Generally, the higher metagenes may also contain meaningful structure but in this article we focus only on the first component of the variation. It may be useful in the future to devise a scheme for extending the analysis to higher metagenes.

Once the activity levels are determined, they can be used in the same kinds of applications as gene expression levels. For example, we might ask which pathways have activity levels that are significantly higher in samples from a case group (e.g., diabetic) than those from a control group. In this article, we mainly perform simple two-group comparisons using *t *statistics (see Methods) but, in principle, any statistical model for the level of expression of individual genes is adaptable to one for activity levels.

### Type 2 diabetes

The data set analyzed by Mootha *et al*. [9] contains gene expression profiles in muscle tissue for each of 17 type 2 diabetics (DM2), 17 subjects with normal glucose tolerance (NGT), and 9 with impaired glucose tolerance (IGT). The gene expression data is available at the Whitehead Institute Center for Genome Research website [16] along with phenotype data including, for example, ages, body size measurements, blood glucose levels after oral glucose tolerance test (OGTT), and other information. More details can be found in Ref. [9].

The calculations of activity levels were done using all 43 gene expression profiles. Using the *t *statistic to compare mean activity levels identified no pathways showing apparently different expression between any of the groups DM2, IGT, and NGT. Specifically, the pathway with the highest significance level, 'Activation of cAMP-dependent protein kinase, PKA (protein kinase A)' (Biocarta) was found in comparing DM2 and NGT (upregulated in DM2); the significance level, however, was calculated to be only *p *= 0.4.

**Figure 3:**
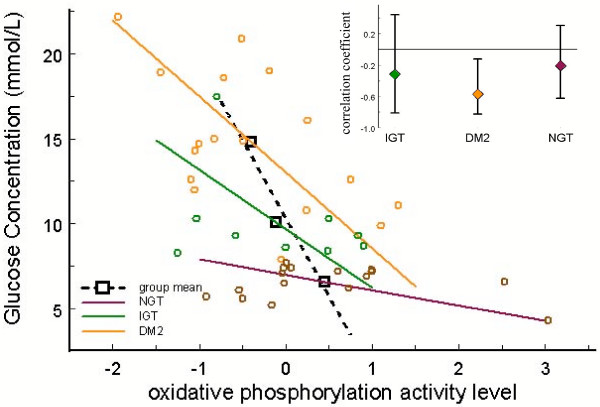
**Negative correlation between oxidative phosphorylation and blood glucose levels after OGTT**. Scatter plot of blood glucose levels 2 hours after OGTT vs. oxidative phosphorylation activity levels. The three subject groups – type 2 diabetic (DM2), normal glucose tolerance (NGT), and impaired glucose tolerance (IGT) – are distinguished by color; solid lines show the first principal component for each group independent of the others. Group means are shown in black squares. The inset shows the 95% confidence intervals for the linear correlation coefficients for each group. Negative correlation between glucose levels and oxidative phosphorylation reaches statistical significance only within DM2 subjects.

We then looked for correlation between activity levels and potentially more informative variables including blood glucose levels after oral glucose tolerance test (OGTT) and VO2max (a measure of maximum oxygen utilization). The most significant pathway identified here was Oxidative Phosphorylation (KEGG); the Oxidative Phosphorylation activity level was found to be significantly negatively correlated with blood glucose concentration 1 hour after OGTT (Pearson correlation *r *= -0.52 and *p *= 0.03). Other pathways found to be correlated with glucose levels 1 hour after OGTT include, in order of significance, Biocarta 'Activation of cAMP-dependent protein kinase, PKA (protein kinase A)' (*r *= +0.47, *p *= 0.06), and KEGG 'ATP synthesis' (*r *= -0.43, *p *= 0.17). The genes in the ATP synthesis pathway, however, are entirely contained within the genes of the KEGG oxidative phosphorylation pathway. The results from these comparisons are summarized in Table 1. A more detailed statistical analysis of the relationship between glucose levels, oxidative phosphorylation, and diabetic status is presented in Fig. 3 and refines the result obtained from the initial analysis.

**Table 1:** Pathways correlated with a type 2 diabetic phenotype. The table shows *p*-values for the three pathways most correlated with blood glucose concentration as measured two hours after an oral glucose tolerance test (OGTT). *r *is the Pearson correlation. Also shown are *p*-values, generally indicating low significance levels, for these pathways determined from *t*-statistic comparisons between DM2 (type 2 diabetic) and NGT (normal glucose tolerance).

	Comparison	
(genes in data set/total genes in pathway) Pathway	glucose after OGTT	DM2 vs. NGT

(96/123) Oxidative phosphorylation	0.031 (*r *= -0.517)	0.565 (down in DM2)
(6/6) Activation of cAMP-dependent protein kinase	0.062 (*r *= +0.474)	0.395 (up in DM2)
(35/40) ATP synthesis	0.166 (*r *= -0.432)	0.855 (down in DM2)

The connection between blood glucose levels and oxidative phosphorylation (and ATP synthesis) seems biologically reasonable. Oxidative phosphorylation makes up the last few steps in the series of reactions leading to the synthesis of ATP from the oxidation of glucose. Unusually high blood glucose levels imply a relatively low activity of this pathway. Protein kinase A (PKA) facilitates the breakdown of glycogen into glucose in skeletal muscle cells [17]; an elevated PKA pathway in skeletal muscle of subjects exhibiting a type 2 diabetic phenotype may then possibly be interpreted as a cellular reaction to glucose starvation.

We note that our results differ slightly from those of Mootha *et al*. [9] who, using GSEA, found a significant (*p *= 0.029) downregulation of oxidative phosphorylation genes in diabetics compared to non. In contrast, we find reasonable significance only in the level of correlation with blood glucose levels after OGTT. This and other differences with GSEA are discussed in a comparison below.

### Effects of smoking on airway epithelia

A recent study examined the effects of smoking on gene expression in airway epithelia [10]. Expression data were obtained from a large number of subjects including former and current smokers, and those who have never smoked. The study identified genes differentially expressed between the different groups and some of the general functional categories represented by these genes. A pathway based analysis can be complementary to the kind already given by drawing attention to groups of genes involved in more specific cellular processes.

We obtained the gene expression data for the smoking study from the Airway Gene Expression Database (AGED) [18]. The data consist of gene expression profiles from 75 subjects including 34 current smokers, 18 former smokers, and 23 subjects who have never been smokers. The data set has undergone some preprocessing steps including normalization and filtering for genes detected on the microarrays. More details can be found at the AGED website [18] and in Ref. [10].

**Figure 4:**
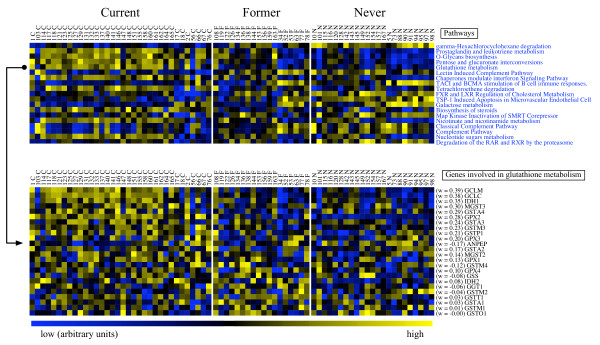
**Expression profiles in airway epithelia of current (C), former (F), and never (N) smokers**. Top: colormap of pathway activity levels for the highest ranking pathways in the comparison between current smokers and never smokers. Bottom: colormap for genes in the KEGG glutathione metabolism pathway. Glutathione is an important anti-oxidant known to be increased in the lungs of smokers. The genes with the highest weights in this pathway, *GCLM *and *GCLC*, encode the subunits of glutamate cysteine ligase (*GCL*), the rate-limiting enzyme in the synthesis of glutathione [24].

We identified pathways most differentially expressed in two-group comparisons using *t *statistics (see Methods). Table 2 shows the top ranking pathways from these comparisons and *p*-values. The top of Fig. 4 shows a colormap of the activity levels for these pathways in the different samples. The bottom of Fig. 4 shows a colormap for the expression levels of the genes in the KEGG glutathione metabolism pathway; colormaps for the other pathways can be viewed at the PLAGE website [11].

**Table 2:** Top pathways identified in comparisons between current (C), former (F), and never (N) smokers. Pathways identified as differentially expressed with *p *< 0.05 using pathway activity levels in comparisons between smokers and non-smokers. The *p*-values were determined using 10,000 random permutations as described in the Methods section. *p *< 0.0001 means no pathway in any of the 10,000 permutations showed higher significance. We note that the change (up or down) is determined, somewhat arbitrarily, by the average expression level captured by the first metagene. Specifically, the pathway is called 'up' if the average of ∑_*i*_*c*_*j*_*w*_*i *_is greater in the first group (e.g., C in 'C vs. N') than in the second. A given pathway, however, will typically have some genes with higher and some with lower mean expression in one group as compared to another.

Comparison	(genes in data set/total genes in pathway) Pathway	*p*	change
C vs. N	(14/45) gamma-Hexachlorocyclohexane degradation	<0.0001	down
	(15/39) Prostaglandin and leukotriene metabolism	<0.0001	up
	(11/24) O-Glycans biosynthesis	<0.0001	up
	(6/21) Pentose and glucuronate interconversions	<0.0001	up
	(24/34) Glutathione metabolism	<0.0001	up
	(3/12) Lectin Induced Complement Pathway	0.0004	down
	(11/19) Chaperones modulate interferon Signaling Pathway	0.0006	up
	(6/15) TACI and BCMA stimulation of B cell immune responses.	0.0044	down
	(3/6) Tetrachloroethene degradation	0.0054	up
	(3/6) FXR and LXR Regulation of Cholesterol Metabolism	0.0062	down
	(4/7) TSP-1 Induced Apoptosis in Microvascular Endothelial Cell	0.0065	down
	(16/28) Galactose metabolism	0.0067	down
	(13/20) Biosynthesis of steroids	0.0164	up
	(7/11) Map Kinase Inactivation of SMRT Corepressor	0.0274	down
	(25/68) Nicotinate and nicotinamide metabolism	0.0279	up
	(4/14) Classical Complement Pathway	0.0314	down
	(6/19) Complement Pathway	0.0364	down
	(9/13) Nucleotide sugars metabolism	0.0369	up
	(3/3) Degradation of the RAR and RXR by the proteasome	0.0396	down

F vs. C	(3/12) Lectin Induced Complement Pathway	0.0068	up
	(13/20) Biosynthesis of steroids	0.0083	down
	(24/34) Glutathione metabolism	0.0403	down

F vs. N	(14/45) gamma-Hexachlorocyclohexane degradation	0.0321	down

We also performed one-way ANOVA using pathway activity levels and found the top pathways from this analysis (those with the smallest *p*-values) to be virtually the same as those identified using *t *statistics.

The pathway most significantly differentially expressed in both current and former smokers as compared to never smokers is 'gamma-Hexachlorocyclohexane degradation' (KEGG). Genes in this pathway and corresponding weights from SVD are given in Table 3. Gamma-Hexachlorocyclohexane, also known as lindane, is a toxic insecticide and the connection with smoking is not at first clear. Several genes in the gamma-Hexachlorocyclohexane degradation pathway, however, have known associations with smoking. The gene with the highest absolute weight, *CYP2A6*, plays a key role in the metabolism of nicotine and a number of studies have indicated an important relationship between *CYP2A6 *and smoking [19,20]. For example, individuals with inactive *CYP2A6 *alleles have been reported to be at lower risk for becoming dependent on cigarettes [21]. Our analysis highlights the potential importance of a cellular process in connection with smoking and further, by the high absolute weight assigned to *CYP2A6*, points to the key role played by *CYP2A6 *in this process.

**Table 3:** Weights for genes in two pathways that show an association with smoking status. SVD weights for genes in pathways identified as significantly differentially expressed between current and never smokers. The gene with highest absolute weight in the gamma-Hexachlorocyclohexane degradation pathway, *CYP2A6*, plays a key role in nicotine metabolism and has been linked to nicotine dependence [21]. *GALNT3*, a gene with relatively high weight in the O-Glycans biosynthesis pathway, initiates mucin-type O-glycosylation [22], suggesting a connection with the increased sputum production observed in smokers. The overall sign of the weights has here been chosen so that a positive weight implies relatively higher expression in current as compared to never smokers.

Pathway	gene	weight
gamma-Hexachlorocyclohexane degradation	*CYP2A6*	-0.42
	*CYP2F1*	-0.38
	*CYP1B1*	0.38
	*CYP2A7*	-0.38
	*ALPL*	0.28
	*CYP2B6*	-0.27
	*CYP4B1*	-0.27
	*CYP2J2*	-0.23
	*PON2*	0.19
	*ACP5*	0.15
	*ACP2*	0.15
	*ACP1*	0.15
	*CYP2C9*	0.09
	*CYP1A2*	-0.02

O-Glycans biosynthesis	*GALNT7*	0.50
	*GALNT1*	0.42
	*GALNT3*	0.42
	*B4GALT4*	0.38
	*GALNT6*	0.28
	*B4GALT1*	0.23
	*GALNT2*	0.21
	*C1GALT1*	0.20
	*OGT*	-0.15
	*B4GALT3*	0.11
	*B4GALT2*	0.05

Other pathways upregulated in current vs. never smokers include metabolism of prostaglandins and leukotrienes (associated with pain response and inflammation), O-Glycans biosynthesis, and glutathione metabolism.

Genes from the O-Glycans pathway are listed in Table 3. Several of these are also within the list of genes identified by Spira *et al*. [10] The present analysis, however, reveals more directly the functional relationship shared by the genes and draws special attention to the role of this pathway in the physiological response to smoking. O-linked glycosylation plays an important role in the production of proteoglycans, some of which are constituents of mucus [17]. In particular, one of the O-Glycans genes *GALNT3 *initiates mucin-type O-glycosylation [22]. It therefore seems plausible that the elevated activity of the O-Glycans pathway is linked to the increased sputum production observed in smokers [23].

Glutathione is an important antioxidant known to be increased in the lungs of smokers [24]. A colormap of the expression levels of the genes in the glutathione metabolism pathway is shown in Fig. 4; the higher expression of many of these genes in the current smokers is clear. The increased expression of glutathione metabolism genes in smokers was also noted by Spira *et al*. [10]. Interestingly, the two genes with the highest weights are *GCLM *and *GCLC*; these genes encode the subunits of glutamate cysteine ligase (*GCL*), which is the rate-limiting enzyme in the synthesis of glutathione [24].

### Comparison with gene-set enrichment analysis

We used GSEA to identify significant pathways in comparisons between the different groups of smokers. The ranking of genes required to evaluate enrichment scores was done, following Ref. [9], using the signal to noise ratio (the absolute value of the difference of the means of the two groups divided by the sum of the within-group standard deviations). For this analysis we used our collection of pathways and complexes. In comparing current and never smokers, the gene-set with the highest enrichment score (ES) was determined to be the KEGG ribosome genes with an ES of 162. However, using 1000 random permutations as described in Ref. [9] to evaluate the significance of this ES yielded a *p*-value of only 0.17. Comparing former and never smokers, GSEA also finds the ribosome gene-set to have the highest ES (304) and with a reasonably high significance level of *p *= 0.047 (1000 permutations). Finally, the comparison between current and former smokers identifies 'Lectin Induced Complement Pathway' (Biocarta) as the pathway with the highest ES (128) but the significance level is very low (*p *= 0.52).

We also used GSEA with our collection of gene-sets to compare DM2 and NGT in the diabetes data discussed above. Here the ribosome gene-set is found to have the highest ES (278) and *p *= 0.073 (1000 permutations). Oxidative phosphorylation has the next highest ES (261) with *p *= 0.092.

It is interesting that in three of our four comparisons, the ribosome gene-set is found to have the highest ES. We noticed that the expression levels for the ribosome genes are very strongly correlated. This can be seen very clearly in colormaps of the expression levels in both the diabetes and smoking data sets (not shown) and is also implied by an unusually large first eigenvalue. For example, in the smoking data set the first metagene for ribosome accounts for over fifty percent of the total variation,  = 0.53. In comparison, this fraction is only 0.23 for Oxidative Phosphorylation (which has 87 genes vs. the ribosome's 80 genes present in the data set) and 0.05 for uncorrelated gaussian noise obtained from averaging over 1000 simulated data sets of the same size as the ribosome. These observations lead us to speculate that GSEA may not effectively distinguish between gene sets that show a consistent difference between groups of samples and sets with genes that are merely strongly coordinately expressed. In the method we have proposed, correlated expression patterns influence the form of the first metagene and the pathway activity levels, but this is treated separately from the question of whether there is a consistent difference between groups.

To summarize, our approach suggests a number of potentially interesting pathways and hypotheses from comparisons between smokers and non-smokers. The results of our analysis seem biologically reasonable and are more consistent with the findings of Spira *et al*. [10] than those obtained by GSEA. As a specific example, we identified glutathione metabolism genes as having significantly (*p *< 0.0001) higher expression levels in current vs. never smokers. This feature was also noted by Spira *et al*. [10] in their original analysis and seems evident in the colormap shown in Fig. 4. GSEA, however, does not find this or any other pathway to be differentially expressed below *p *= 0.05 between current and never smokers.

## Conclusion

We have introduced a method for analyzing gene expression data in terms of a collection of predefined pathways and complexes. In summary, the approach quantifies the level of activity of each pathway within each sample and uses the activity levels as the basis for making comparisons. Mootha *et al*. [9] previously demonstrated that looking at expression data in terms of predefined pathways can provide valuable insights not easily attainable by methods more focused on individual genes and we feel that the results in this article reinforce their view.

The main applications we have presented are simple two-group comparisons. We suggest that pathway activity levels may also be useful in other areas, for example, as variables in models for gene regulation (e.g., Bayesian networks [25]), for clustering and classification [15,26], and in computational efforts to discover novel gene-pathway associations. One possibility is to use SVD on the activity levels themselves to determine 'metapathway' signatures for the status of some disease following the approach described in Ref. [15]. There are a number of potential advantages to be gained in these and other cases. For one, features defined by groups of genes will tend to be more robust in the face of variation at the level of individual genes. In addition, the features here (pathways) are based on a large amount of prior biological knowledge and so are potentially more directly biologically meaningful.

## Methods

### Gene sets

As in GSEA, the basis for our analysis is a classification of genes into known pathways and complexes. Here, we use about 400 pathways and complexes characterized on the Biocarta and KEGG (Kyoto Encyclopedia of Genes and Genomes) websites [12,13]. The KEGG pathways mainly include processes related to metabolism and biosynthesis (e.g., Fatty acid metabolism and Ubiquinone biosynthesis). Those on Biocarta cover a wider variety of cellular processes including a large number of immune signaling pathways (Toll-like receptor pathway, B-cell receptor signaling pathway, complement pathway), as well as metabolic and biosynthetic pathways. Both collections also include complexes such as the T-cell receptor and ribosome and broad gene categories such as cytokines. The KEGG and Biocarta pathways continue to expand and evolve. The versions used for this article are available at the PLAGE website [11].

### Pathway activity levels

Our analysis starts by quantifying, in each sample, the level of 'activity' of each pathway. As described in the Results and discussion section, we define the activity level of a pathway in a given sample as the 'level' of expression of a certain metagene in that sample, i.e., the level of the first metagene from the SVD (e.g., Ref [27]) of the matrix of expression levels. In this section we provide the details and motivation behind this choice.

We begin by standardizing the gene expression levels to have zero mean and unit variance over samples. For each pathway, we form a matrix *Y *(rows = genes, columns = samples) containing the standardized expression levels from all samples but for the genes in that pathway only. We write the singular value decomposition of *Y *as

*Y *= *WDC*.     (1)

Here the columns of the matrix *W *are the orthonormal (*W*^⊤^*W *= *I*, the identity matrix) eigenvectors or metagenes of *Y*, *D *is a diagonal matrix containing the associated eigenvalues, and each column of *C *is a vector of coefficients for one of the samples indicating the level of each metagene in the sample. The rows of *C *are also orthonormal (*CC*^⊤ ^= *I*). Assume the eigenvalues are ordered from highest to lowest going down the diagonal of *D*. The first metagene **w **– that associated with the largest eigenvalue – is then the first column of *W*. We write its eigenvalue as *λ *and the associated coefficients (first row of *C*) as *c*_*j*_.

The activity level of a pathway in a given sample *j *is taken as the coefficient *c*_*j *_for the first metagene. It follows also from the orthonormality of the columns of *W *and rows of *C *that



That is, the activity level *c*_*j *_can also be regarded (up to a non-essential scale factor) as a weighted sum of the standardized expression levels of the individual genes, the weights being given by the first metagene **w**.

One motivation for using the first metagene in SVD is that the resulting combination of activity levels and weights specifies an optimal approximation to the matrix *Y *(i.e., accounts for the main component of the variation in the data). Specifically, assume the following statistical model for the expression levels

*y*_*ij *_= *α*_*i*_*χ*_*j *_+ *ε*_*ij *_    (3)

where the vector ***χ ***is constrained to have unit norm and the *ε*_*ij *_are independent Gaussian random variables. The estimates for ***α ***and ***χ ***that minimize the sum of the squared errors are just the first metagene scaled by its eigenvalue, *λ***w**, and the associated vector of activity levels **c**, respectively. The approximation of a set of expression profiles using the first metagene is illustrated in Fig. 2.

A useful fact about the first eigenvalue is that its square is a measure of the amount of variation accounted for by the first metagene. Specifically, with *n*_*g *_= number of genes and *n*_*s *_= number of samples, the total amount of variation in the data is  (recall, the expression levels are standardized so that ) and the variation remaining after subtracting off the profile described by the first metagene is ∑_*ij*_(*y*_*ij *_- *λw*_*i*_*c*_*j*_)^2 ^= *n*_*g*_(*n*_*s *_- 1) - *λ*^2^.

### Evaluating significance

In this article, we mainly perform pairwise comparisons to identify pathways for which the mean activity level in one group (e.g., diabetic) is significantly different from that in the other (non-diabetic). To accomplish this we calculate a *t *statistic for each pathway:



where *A *and *B *are labels for the groups, *n*_*A *_is the number of samples in group *A*, and *μ*_*A *_and *V*_*A *_are the mean and variance of the activity level in *A *(similarly for group *B*). To determine a fair measure of significance in an analysis of this kind, it is important to account for the fact that we are testing a large number of hypotheses [28]. For this purpose, we perform a large number (10,000 for this article) of comparisons by randomly permuting the sample labels and for each permutation recording the *t *statistic for the most significant pathway (the maximum t-statistic) identified using the permuted labels. Our *p*-values are computed with reference to the maximum t-statistic, i.e., we calculate a *p*-value by taking the fraction of the maximum t-statistics that exceed the *t*-statistic.

This basic approach was also used by Mootha *et al*. [9] to determine the significance of the ES (enrichment score) for a pathway except that the comparison there is with maximum enrichment scores. Generally, other statistics can be used in similar fashion. For example, to evaluate the significance of the level of correlation of activity levels with blood glucose concentration in the diabetes data, we determined the minimum and maximum (Pearson) correlations between activity levels and randomly permuted glucose concentration values in each of 10,000 permutations. *p*-values were obtained as the fraction of these extremal correlation values that were stronger (higher if *r *> 0 and lower if *r *< 0) than the value obtained using the correct ordering of glucose levels.

## Availability and requirements

The SVD-based pathways analysis method has been implemented in a web program [11] called PLAGE (pathway level analysis of gene expression). PLAGE will run through standard web browsers.

## Authors' contributions

JT, JL, and TBK all contributed to the development of the method and the writing of the manuscript. JT wrote the web program.
